# Comparison between optical coherence tomography angiography and immunolabeling for evaluation of laser-induced choroidal neovascularization

**DOI:** 10.1371/journal.pone.0201958

**Published:** 2018-08-09

**Authors:** Kazuki Nakagawa, Haruhiko Yamada, Hidetsugu Mori, Keiko Toyama, Kanji Takahashi

**Affiliations:** Department of Ophthalmology, Kansai Medical University, Hirakata, Osaka, Japan; University of Florida, UNITED STATES

## Abstract

This study aimed to investigate the differences between images obtained by optical coherence tomography angiography (OCTA) with those from immunohistochemical labeling of laser-induced choroidal neovascularization (CNV) in a mouse model. CNV was induced by laser photocoagulation (GYC-2000, NIDEK; wavelength 532 nm) in the left eyes of 10 female C57BL/6J mice aged 6 weeks. The laser parameters included a 100-μm spot, 100-ms pulse duration and 200-mW incident power to rupture Bruch’s membrane. OCT and OCTA CNV images were obtained using the RS-3000 Advance (NIDEK) 5 days post-laser photocoagulation. After OCTA imaging, the isolated choroid/retinal pigment epithelium complexes were fluorescently labeled with CD31 (an endothelial cell marker), platelet-derived growth factor receptor β (PDGFRβ, a pericyte-like scaffold marker), α-smooth muscle actin (α-SMA) and collagen I. Area measurements of the lesions obtained by enface OCTA were compared with immunolabeled CD31+ CNV lesions in choroid flat-mounts. We also examined structural correlations between the PDGFRβ+ pericyte-like scaffold and OCTA images. Laser-induced CNV was clearly detected by enface OCTA, appearing as a hyperflow lesion surrounded by a dark halo. Area measurements of the CNV lesion by immunolabeling were significantly larger than those obtained by enface OCTA (*p* = 0.006). The CNV lesion beneath the periphery of the pericyte-like scaffold was not clearly visible by enface OCTA due to the dark halo; however, the lesion was detectable as blood flow by cross-sectional OCTA and was also highly labeled by CD31. The periphery of the pericyte-like scaffold appeared to develop into subretinal fibrosis and this region was rich in myofibroblasts. Enface OCTA was unable to detect the entire area of laser-induced CNV in mice, with an undetectable portion located beneath the fibrotic periphery of the pericyte-like scaffold. Due to this OCTA fibrosis artifact, OCTA imaging has limited potential for accurately estimating CNV lesions.

## Introduction

Optical coherence tomography angiography (OCTA) is a novel imaging tool which allows the visualization of the retinal and choroidal vasculature. This method is based on split-spectrum amplitude-decorrelation angiography (SSADA) and detects blood flow as the motion of erythrocytes [1]. Because of its noninvasive aspects (OCTA does not require the use of dye injection as in fluorescein angiography [FA] or indocyanine green angiography [ICGA]), this new method has been successfully applied in daily clinical ophthalmological practice. Moreover, OCTA has the unique capability of layer analysis and repeatable quantitative analysis. Some studies have reported that OCTA could be used to detect choroidal neovascularization (CNV) and evaluate therapeutic responses in exudative age-related macular degeneration (AMD) [2,3]. However, OCTA has undesirable imaging artifacts [4]. Thus, it is important to precisely explore and understand the benefits and limitations of OCTA for accurate assessment of CNV.

Laser-induced CNV is a reliable animal model for exudative AMD and consists of three stages [5]. The first stage (at 0–3 days post-laser injury) is an inflammatory response which initiates a growth phase. The second stage (at 3–5 days post-laser injury) is the development of CNV. CNV size reaches a maximum at 5 days post-laser injury. The third stage (after 5 days post-laser injury) is the regression of CNV. Both the balance of damage to the choroid/Bruch’s membrane/retinal pigment epithelium (RPE) complex and the ability of RPE cells to encircle the damaged area determine the evolution of CNV [5,6]. Recently, He et al. [7] suggested that the pericyte-like scaffold, also referred to as the myofibroblastic scaffold, forms during the early wound healing response before the infiltration of CD31+ endothelial cells into the site of laser injury, and expresses high levels of IL-1β as a proangiogenic stimulus. According to their studies, M2 type macrophages infiltrate the site of injury within the first 24 to 40 hours, followed by the appearance of a pericyte-like scaffold strongly expressing smooth muscle actin (SMA) in the CNV lesion 60 hours after injury. Endothelial cells then form the CNV lesion. It is thought that the pericyte-like scaffold covers and demarcates the site of angiogenesis and simultaneously leads a colony-like CNV lesion. He et al. also suggested that the pericyte-like scaffold is not derived from transdifferentiated RPE cells, but from cells of the underlying choroid, such as pericyte-like cells [7,8]. These pericyte-like cells show intense expression of SMA and platelet-derived growth factor receptor β (PDGFRβ). Because the RPE cells adjacent to the site of laser injury are activated and express SMA in response to laser injury, PDGFRβ is a suitable target for specifically evaluating the pericyte-like cells. Moreover, inhibition of PDGFRβ+ cell proliferation prior to neovessel formation suppressed scaffold formation and neovascularization [8].

As described above, the chronological changes in the histology of laser-induced CNV have been well characterized. However, the correlation between the histological characteristics of CNV and OCTA images is poorly understood. In order to accurately assess CNV using OCTA, it is necessary to understand how the histological characteristics influence OCTA imaging. In this study, we imaged laser-induced CNV at 5 days post-laser photocoagulation in mice by OCTA and compared the area of the CNV lesion obtained by OCTA imaging with that obtained by immunolabeling of choroidal flat-mounts. We also evaluated the correlation between the histological characteristics of the pericyte-like scaffold and OCTA images.

## Materials and methods

### Animals

All animal experiments followed the guidelines of the ARVO Statement for the Use of Animals in Ophthalmic and Vision Research and were approved by the Animal Care Committee of Kansai Medical University (approval number: 17–013). Wild-type (WT) C57BL/6J mice were purchased from CLEA Japan (Tokyo, Japan). A total of 10 female mice aged 6 weeks were used for this study. All mice were kept in pathogen-free plastic cages with 12 hour light-dark cycles and had continuous free access to water and food. All plastic cages, water and bedding feed were purified before use. For all procedures, anesthetization was achieved by intraperitoneal injection of 90 mg/kg ketamine hydrochloride (Daiichi Sankyo Co., Tokyo, Japan) and 40 mg/kg xylazine (Bayer, Berlin, Germany); pupils were dilated with topical 0.5% tropicamide and 0.5% phenylephrine (Santen Pharmaceutical, Osaka, Japan).

### Laser-induced CNV model

**Fig 1:**
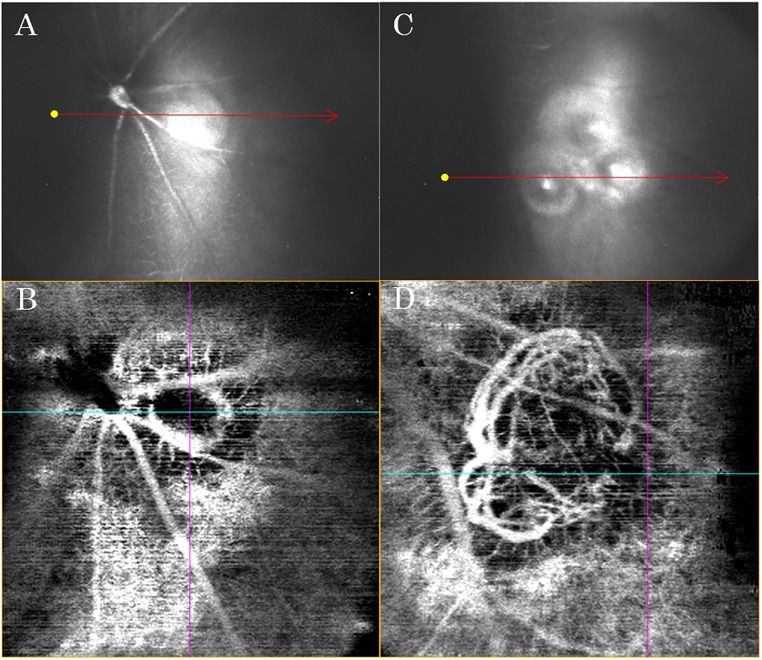
Alternative method for laser-induced CNV irradiation. (A) Near-infrared reflectance (NIR) image obtained immediately after laser photocoagulation of one spot. (B) Enface OCTA image at 5 days post-laser photocoagulation (area measuring 900×900 μm). (A) and (B) are the same eye. (C) NIR image obtained immediately after laser photocoagulation of three spots. (D) Enface OCTA image at 5 days post-laser photocoagulation (area measuring 900×900 μm). (C) and (D) are the same eye. Note that in the three-spot laser photocoagulation irradiation, the spots overlapped each other to create a larger disruption of the choroid/Bruch’s membrane/RPE complex, resulting in a larger CNV lesion that is easier to observe and evaluate in detail by enface OCTA (D) than one-spot laser photocoagulation.

CNV was induced by laser photocoagulation (GYC-2000; NIDEK Co, Japan; wavelength 532 nm) attached to a slit-lamp delivery system (Carl Zeiss SL 130, Jena, Germany) in the left eye of each mouse, with a spot size of 100-μm, a 100-ms pulse duration and a 200-mW incident power applied to the posterior pole to rupture Bruch’s membrane. If the laser photocoagulation irradiated only one spot, the CNV lesion was too small to be observed in detail by OCTA (Fig 1A and 1B). However, when laser photocoagulation irradiated three overlapping spots to enlarge the disruption of the choroid/Bruch’s membrane/RPE complex, the CNV lesion was large enough to be observed in detail meby OCTA (Fig 1C and 1D). Thus, we used three spots per eye for the procedure in this study.

### Optical coherence tomography angiography imaging

OCT and OCTA were performed using the RS-3000 Advance (NIDEK Co, Japan). A mouse-specific adapter with a power of 10 diopters was installed for OCT and OCTA and a platform was built to stably position the mice in front of the RS-3000 Advance, similar to previous studies [9–11]. This instrument has an A-scan rate of 53,000 scans per second, and uses a light source centered at 880 nm. For OCTA, 8 linear B-scans were acquired from 256 cross sectional locations and retinal areas measuring 900×900 μm were centered on the CNV lesion and scanned focusing on the retinal vasculature. OCT scans, which have a linear measurement of 900 μm on the retina, were acquired at 50 frames per image. The left eye of each mouse wore a plain hard contact lens (UNICON, Osaka, Japan) to prevent drying of the cornea and unwanted cataract formation. Laser-induced CNV images were acquired 5 days post-laser photocoagulation. We obtained 3D enface OCTA images between the deep retina and choroid using the semi-automatic mode with some modification to the layer settings provided by RS-3000 Advance to the first author.

### Choroidal flat mount preparation for immunofluorescence imaging

Immediately after in vivo imaging by OCTA, deeply anesthetized mice were sacrificed by intracardial perfusion with PBS after thoracotomy. After PBS perfusion, eyes were enucleated and fixed in 2% paraformaldehyde/PBS for 10 minutes. The choroid/RPE complex was isolated and permeabilized with 0.5% Triton X-100, 20% DMSO and 0.6% Block Ace blocking reagent (DS Pharma Biomedical, Osaka, Japan) in PBS for 3 hours at room temperature [9] and subsequently used for immunolabeling. The following antibodies were used for whole-mount immunolabeling of the choroid: i) anti-CD31 monoclonal antibody (rat anti-mouse CD31, MEC 13.3, 1:15; BD Biosciences Pharmingen, San Diego, CA) to detect blood vessels with Alexa Fluor-488 secondary antibody (1:500); ii) anti-PDGFRβ monoclonal antibodies (rabbit anti-mouse PDGFRβ, ab32570, 1:500; Abcam, Cambridge, MA) to detect the pericyte-like scaffold with Alexa Fluor-568 secondary antibody (1:500). In addition, a monoclonal antibody against SMA (mouse anti-mouse α-SMA, ab7817, 1:2000; Abcam, Cambridge, MA) with Alexa Fluor-488 secondary antibody (1:500) and a polyclonal antibody against collagen I (rabbit anti-mouse collagen I, ab34710, 1:200; Abcam, Cambridge, MA) with Alexa Fluor-647 secondary antibody (1:500) were used to compare with an anti-PDGFRβ monoclonal antibody (rat anti-mouse PDGFRβ, CD140b, 1:100; Thermo Fisher Scientific) with Alexa Fluor-568 secondary antibody (1:500). The choroid was incubated in primary antibody solution at 4°C for 2 days. After washing in PBS, the choroid was incubated for 4 hours at room temperature in secondary antibody solution [9]. Four cuts were made from the edge toward the center after thoroughly washing the choroid, which was then flattened and mounted with ProLong Gold anti-fade reagent (Life Technologies, Eugene, OR, USA) and cover-slipped. Frozen sections cut to 8-μm thickness with a cryostat (Leica CM3050S^®^; Leica Biosystems, Nuβloch, Germany) were also processed for staining (for supporting information). The CNV was stained with an anti-CD31 monoclonal antibody (rat anti-mouse CD31, MEC 13.3, 1:50; BD Biosciences Pharmingen, San Diego, CA) and an Alexa Fluor-488 secondary antibody (1:500). The pericyte-like scaffold was stained with an anti-PDGFRβ monoclonal antibody (rabbit anti-mouse PDGFRB, ab32570, 1:500; Abcam, Cambridge, MA) and an Alexa Fluor-568 secondary antibody (1:500). The choroid flat-mounts and the frozen sections were visualized with a fluorescence microscope (BX51; Olympus Corporation, Tokyo, Japan). Immunolabeled images were obtained with a charge-coupled device camera and captured with Basler (Ahrensburg, Germany) microscopy software.

### Quantification of CNV area

**Fig 2:**
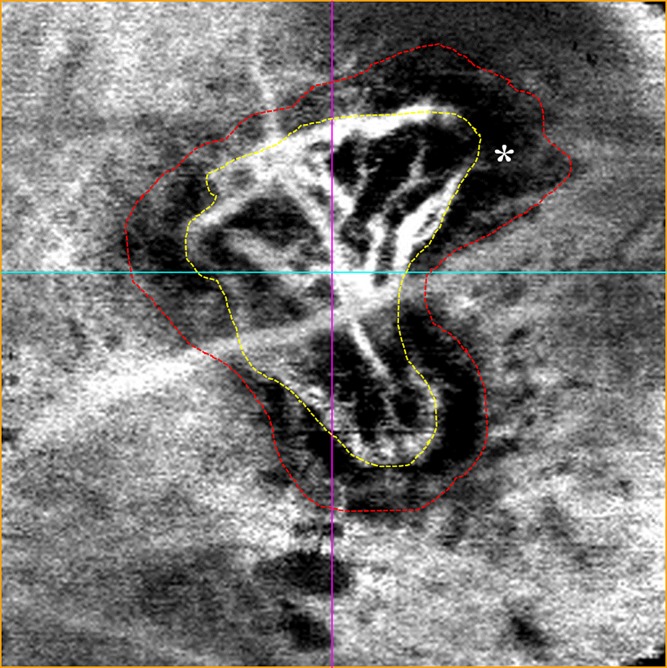
Enface OCTA image of laser-induced CNV at 5 days post-laser photocoagulation. CNV was visualized as a hyperflow lesion surrounded by a dark halo, indicated by the asterisk (*). We measured the CNV lesion within the yellow-dotted line. The dark halo comprising the CNV lesion was measured within the red-dotted line (OCTA measurement area: 900×900 μm).

To calculate the area of the CNV lesion by either OCTA imaging or CD31+ immunolabeling, two masked graders (HM and KT) independently determined the outlines of the CNV lesions using the same exported images. Basler microscopy software was used to quantify these regions in the OCTA and immunolabeled images. The results of the two graders were compared and the correlation coefficients were calculated. In addition to assess the CNV lesion, the dark halo comprising the CNV lesion in OCTA images and the PDGFRβ+ pericyte-like scaffold area determined by immunolabeling were calculated and compared in the same manner. The evaluation method for OCTA images is illustrated in Fig 2.

### Statistical analysis

The areas measured by two masked independent graders were analyzed by correlation coefficient and Bland-Altman plot [10]. Results with non-normal distribution are expressed as the median (interquartile range). A Kruskal-Wallis test was performed using JMP^®^ (SAS Institute Inc., Caly, NC, USA). A *p* value of less than 0.05 was considered significant.

## Results

### Imaging of laser-induced CNV in mice by OCTA

**Fig 3:**
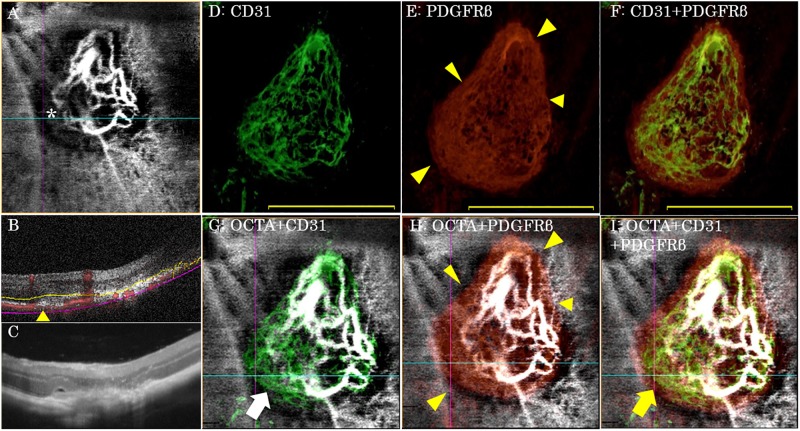
Representative OCT, OCTA and immunolabeling images of laser-induced CNV at day 5 post-laser photocoagulation. (A) By enface OCTA, laser-induced CNV was observed as a hyperflow lesion surrounded by a dark halo (indicated by an asterisk *; measurement area: 900×900 μm). (B) By cross-sectional OCTA imaging (corresponding to the horizontal blue line in Fig 3A) blood flow was detected between the deep retina and choroid (measurement width: 900 μm). (C) By OCT, laser-induced CNV and the pericyte-like scaffold appeared as a subretinal hyper-reflective lesion (measurement width: 900 μm). (D) Laser-induced CNV lesions were stained with CD31. (E) The pericyte-like scaffolds were stained with PDGFRβ. The periphery of the pericyte-like scaffold appeared to develop into subretinal fibrosis (yellow arrowhead). (F) Immunohistochemistry for CD31 merged with the PDGFRβ image. Neovascularization did not extend beyond the pericyte-like scaffold. (G) Enface OCTA image merged with the CD31 immunohistochemistry. The CNV lesion, which was undetectable by enface OCTA imaging, was apparent by immunolabeling (white arrow). (H) Enface OCTA image merged with PDGFRβ immunohistochemistry. The periphery of the pericyte-like scaffold, which developed into subretinal fibrosis, corresponded with the dark halo lesion visible by enface OCTA (yellow arrowhead). (I) Triple-merged image of enface OCTA, CD31, and PDGFRβ immunohistochemistry. Note that the CNV lesion, which was undetectable by enface OCTA imaging, was detectable by immunolabeling (white arrow in Fig 3G). This portion of the CNV lesion, at the periphery of the pericyte-like scaffold, is within the lesion area comprising the dark halo by enface OCTA (yellow arrowhead in Fig 3I). Within this dark halo, blood flow could be detected between the deep retina and the choroid by cross-sectional OCTA (yellow arrowhead in Fig 3B). Scale bar, 500 μm (D-F).

Five days after laser photocoagulation, laser-induced CNV was clearly detected by OCTA (Figs 2 and 3A). CNV was visualized as a hyperflow lesion surrounded by a dark halo by enface OCTA (Figs 2 and 3A). Blood flow was detected between the deep retina and choroid by cross-sectional OCTA (Fig 3B). Laser-induced CNV and the pericyte-like scaffold appeared as a subretinal hyper-reflective lesion by OCT (Fig 3C). A representative OCT image of WT C57BL/6J mouse is shown in S1 Fig.

### Immunolabeled imaging of flat-mounted choroid preparations of laser-induced CNV

**Fig 4:**
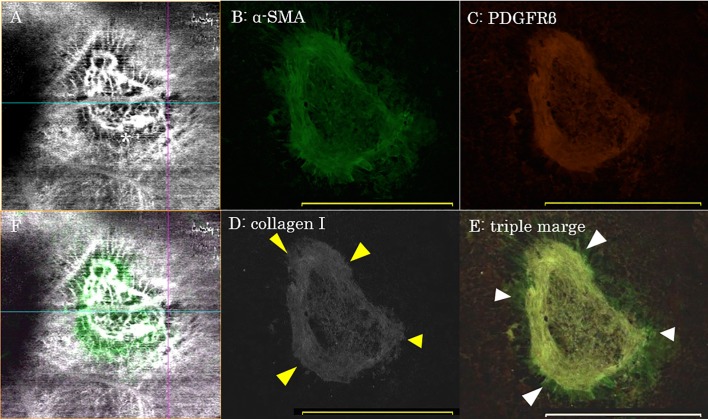
Images from OCTA and immunolabeling experiments in laser-induced CNV at 5 days post-laser photocoagulation. (A) By enface OCTA, laser-induced CNV was observed as a hyperflow lesion surrounded by a dark halo (*; areas measured 900×900 μm). (B-D) The pericyte-like scaffold was stained with α-SMA (B), PDGFRβ (C), and collagen type I (D; shown in gray scale). The periphery of the pericyte-like scaffold appeared to develop into subretinal fibrosis (yellow arrowhead). (E) Merged image of immunohistochemistry for α-SMA, PDGFRβ and collagen type I. The region outside of the pericyte-like scaffold was only stained by α-SMA (white arrowhead). (F) Merged enface OCTA image and immunohistochemistry of the α-SMA positive pericyte-like scaffold. Both outside of and at the periphery of the pericyte-like scaffold, α-SMA+ staining correlated with the dark halo lesion visualized by enface OCTA imaging. Scale bar, 500 μm (B-E).

Five days after laser photocoagulation, new formation of CD31+ vessels (Fig 3D) and a PDGFRβ+ pericyte-like scaffold (Fig 3E) could be clearly observed at the site of laser application. Neovessel formation did not extend beyond the pericyte-like scaffold (Fig 3F), which was positively stained by α-SMA, collagen I and PDGFRβ (Fig 4B–4D). In contrast, the region outside of the pericyte-like scaffold was only stained by α-SMA (white arrowhead in Fig 4E). At the periphery of the pericyte-like scaffold, the development of subretinal fibrosis was apparent (yellow arrowhead in Fig 4D). In order to assess the positional relationship between CNV and the pericyte-like scaffold, frozen sections from laser-induced CNV samples were prepared. The CNV lesions, which were fluorescently labeled with CD31, appeared to be present within the pericyte-like scaffold (PDGFRβ+ region; S2 Fig).

### Comparison of OCTA images with fluorescent images of immunolabeled choroidal flat-mounts in laser-induced CNV

The CNV lesions, which were undetectable by enface OCTA imaging, could be detected by imaging the immunolabelled region (white arrow in Fig 3G). This CNV region was located at the periphery of the pericyte-like scaffold and within the area that appeared as a dark halo lesion by enface OCTA (yellow arrowhead in Fig 3I). In the dark halo lesions, blood flow was detected between the deep retina and choroid by cross-sectional OCTA imaging (yellow arrowhead in Fig 3B); however, the neovascular network could not be detected by enface OCTA. The tissue at the periphery of the pericyte-like scaffold was α-SMA+/collagen I+ indicating the development of fibrosis, and this area corresponded with the dark halo lesion visualized by enface OCTA (Fig 4F).

### Comparison of the CNV area between OCTA and immunolabeling

**Fig 5:**
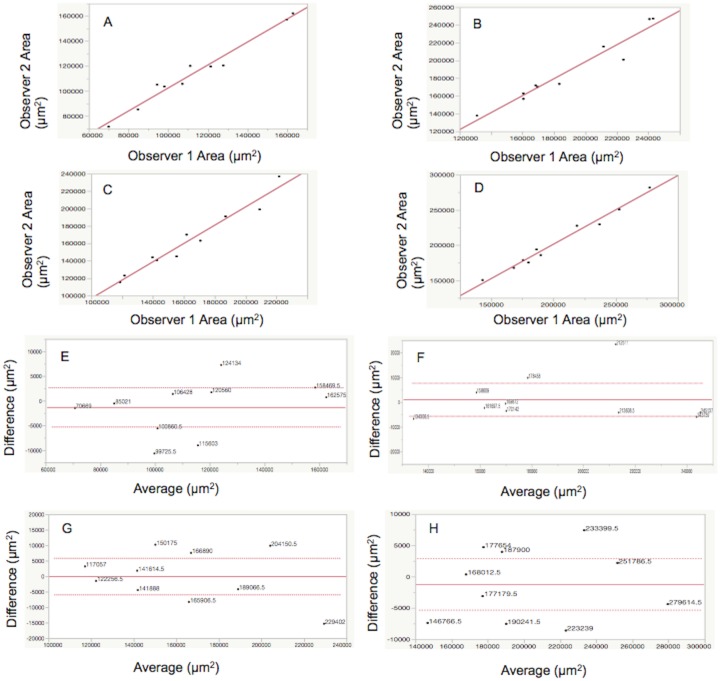
Inter-grader reliability (A-D) and a Brand-Altman plot (E-H) of the area measurements from OCTA and immunolabeling. (A and E) CNV lesion by OCTA. (B and F) dark halo comprising CNV lesion by OCTA. (C and G) CD31+ CNV lesion by immunolabeling. (D and H) PDGFRβ+ pericyte-like scaffold by immunolabeling. The *r*^2^value was 0.97 (A: CNV lesion by OCTA), 0.94 (B: dark halo comprising CNV lesion by OCTA), 0.95 (C: CD31+ CNV lesion by immunolabeling) and 0.98 (D: PDGFRβ+ pericyte-like scaffold by immunolabeling). A Bland-Altman plot showed agreement between the measurements of the two graders (E-H). No particular trend was found in this plot suggesting that there was no systematic bias.

**Fig 6:**
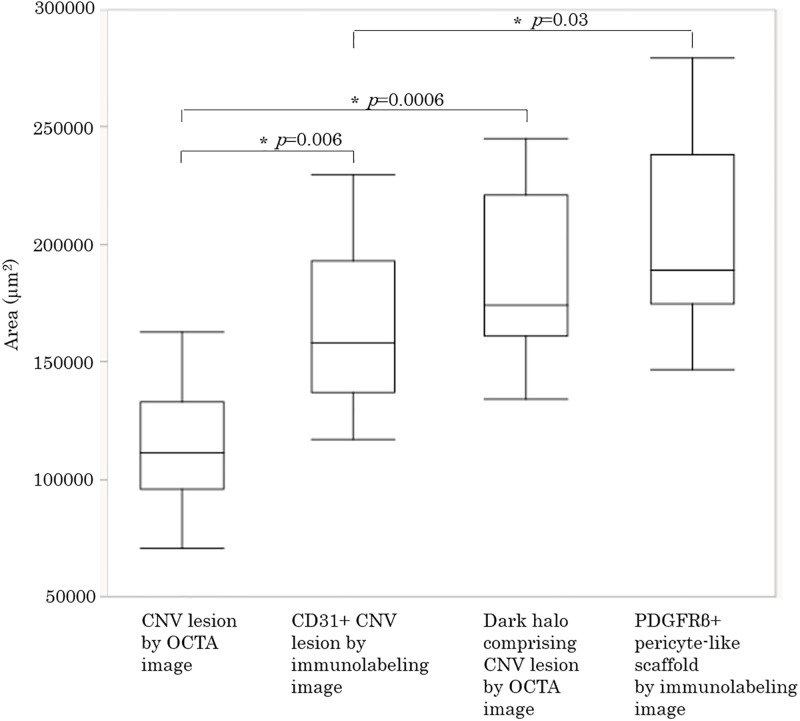
Comparison of area measurements from OCTA and immunolabeled images. Median (interquartile range). Kruskal-Wallis test with a significance level of 0.05 (*).

To determine the comparability of the areas visualized by histological assessment versus OCTA, measurements of the CNV lesions determined by OCTA imaging were compared with measurements obtained by imaging the CD31+ immunolabeled lesions. In addition to evaluating the differences in CNV size, measurements of the dark halo comprising the CNV lesion in OCTA images and area measurements of the immunolabeled PDGFRβ+ pericyte-like scaffold were investigated. The areas measured by two masked independent graders were plotted against each other (Fig 5A–5D). The measured *r*^2^ values for these data were 0.97 (CNV lesion by OCTA), 0.94 (dark halo comprising CNV lesion by OCTA), 0.95 (CD31+ CNV lesion by immunolabeling) and 0.98 (PDGFRβ+ pericyte-like scaffold by immunolabeling). Thus, there was good correlation between the area measurements of the two graders. Furthermore, a Bland-Altman plot of the area measurements was created, with the difference in grader measurements on the *y*-axis and the average grader measurements on the *x*-axis (Fig 5E–5H). No obvious trend was found in this plot, suggesting that there was no systematic bias. The dashed lines in Fig 5E–5H indicate the 95% confidence interval for the difference in measurements, which ranged from -5310 to 2618 (CNV lesion by OCTA), -5705 to 7635 (dark halo comprising CNV lesion by OCTA), -6009 to 5819 (CD31+ CNV lesion by immunolabeling) and -5347 to 2870 (PDGFRβ+ pericyte-like scaffold by immunolabeling). Given the lack of apparent bias and good agreement between the graders, we determined that averaging the graders’ measurements would be suitable for our analysis. A comparison of the measured areas between OCTA images and those obtained by immunolabeling is shown in Fig 6. Area measurements of the CD31+ CNV lesions determined by immunolabeling were significantly larger than those from enface OCTA imaging (*p* = 0.006). Area measurements of the immunolabeled PDGFRβ+ pericyte-like scaffold were significantly larger than those of the CD31+ CNV lesion (*p* = 0.03). Area measurements of the immunolabeled PDGFRβ+ pericyte-like scaffold tended to be larger than those of the dark halo comprising CNV lesion by enface OCTA; however, this difference was not statistically significant (*p* = 0.38).

## Discussion

OCTA, a novel imaging tool, has been used in daily ophthalmological practice and has become indispensable for assessing exudative AMD. However, OCTA has the potential to generate undesirable imaging artifacts [4] which may lead to overestimating or underestimating CNV lesions in exudative AMD. Projection artifacts are one of the most critical artifacts which can affect evaluation of OCTA images. This artifact results in vessels that may appear deeper within the eye than they actually are [4]. These projection artifacts may result in overestimation of the CNV lesion. Alternatively, shadowing is an attenuation of signal behind an absorbing or scattering opacity or obstruction [4]. Shadowing artifacts may lead to false-negative flow and result in the underestimation of CNV lesions. Media opacities, pigment in the RPE and fibrosis also have the potential to cause shadowing. Although there have been some reports of OCTA imaging in laser-induced CNV, these studies did not compare the OCTA images with histological markers other than CD31 or evaluate the presence of OCTA artifacts [9–11]. In this study, we not only compared CNV lesions, but also evaluated the dark halo lesions between OCTA images and those obtained from immunolabeled samples. Our results will be helpful in eliminating OCTA artifacts and more accurately evaluating OCTA images.

Laser-induced CNV has been shown to be a reliable animal model for exudative AMD. As previously reported [9–11], laser-induced CNV in mice could be clearly detected by OCTA at 5 days post-laser photocoagulation in the second stage of CNV development, characterized by new-vessel formation (Fig 3A). The dark halo around the CNV lesion could also be observed by enface OCTA (Fig 3A) and corresponded with the periphery of the pericyte-like scaffold that had developed fibrosis (Fig 3H). In the dark halo lesion, the CD31-labeled region comprising the CNV was undetectable by enface OCTA. The size of the CD31+ CNV lesions by immunolabeling was significantly larger than those of the CNV lesion by enface OCTA (Fig 6). Although it is possible that the CNV lesions are artificially enlarged during the preparation of the choroid as a flat-mount, we assume that the enface OCTA images of laser-induced CNV are actually underestimated due to shadowing artifacts caused by the dark halo which results from subretinal fibrosis of the pericyte-like scaffold. In previous study, Shah RS et al. reported that laser-induced CNV area measurements taken from the isolectin B_4_ stained flatmounts tended to be larger than those of OCTA images at 5 and 7 days post-laser photocoagulation without statistically significant, because isolectin B_4_ stained not only endothelial cells of the perfused blood vessels but also those of the nascent vessels [10]. It is also thought that slow blood flow is a major cause of underestimation of laser-induced CNV by OCTA due to the inability of OCTA to detect slow blood flow [4]. However, we assume that slow blood flow does not lead to underestimation of laser-induced CNV by OCTA because in the dark halo lesions, blood flow was detected between the deep retina and choroid by cross-sectional OCTA imaging (yellow arrowhead in Fig 3B). Moreover, El Ameen et al. reported that the dark ringed area observed in OCTA was present around the CNV lesion in exudative AMD with type 2 CNV and this area corresponded to the dark ring in the early phase of FA [12]. Because type 2 CNV often involves subretinal fibrosis, OCTA may underestimate type 2 CNV due to artifactual shadowing in a clinical setting.

The pericyte-like scaffold is also referred to as the myofibroblastic scaffold [7]. Myofibroblasts are mesenchymal cells that express SMA, PDGFRβ and collagen type I and III [7,8,13–15]. These cells contribute to the formation of fibrosis and play important roles in corneal stromal wound healing, anterior subcapsular cataract, proliferative vitreoretinopathy, proliferative diabetic retinopathy and exudative AMD [16–19]. Myofibroblasts are derived from an epithelial or endothelial-mesenchymal transition, bone marrow-derived fibroblasts and tissue local fibroblasts [15,20,21]. In the laser-induced CNV model, the pericyte-like scaffold is not derived from transdifferentiating RPE cells but from cells of the underlying choroid, such as pericyte-like cells [7]. In this study, the pericyte-like scaffold was stained not only with PDGFRβ but also with α-SMA and collagen I (Fig 4). This suggests that the scaffold is composed of myofibroblasts that will later develop into subretinal fibrosis. Measurements of the pericyte-like scaffold area based on immunolabeling tended to be larger than the measurements of the dark halo comprising CNV lesion by OCTA, although this difference was not significant (*p* = 0.38). It is possible that during tissue processing the pericyte-like scaffold could become extended during the choroid flat-mounting. However, it is also possible that the dark halo visualized by OCTA imaging does not precisely correlate with the periphery of the pericyte-like scaffold. The peripheral border of the dark halo lesion by OCTA imaging was often blurry (Fig 4A) while the border of the pericyte-like scaffold was sharp (Figs 3E, 4C and 4D). As a previous study reported [8], the RPE cells adjacent to the site of laser injury are activated and express α-SMA (Fig 4B and white arrowhead in Fig 4E). While there is some accumulation of activated RPE cells around the periphery of the pericyte-like scaffold corresponding to the dark halo lesion in OCTA images (Fig 4F), it is possible that the OCTA artifact resulting from activated RPE cells did not affect the estimation of CNV size, as the CD31+ CNV region never extended to the PDGFRβ+ pericyte-like scaffold (Fig 3F).

We acknowledge that this study has several limitations. Because of the small number of samples, it was not possible to fully determine the reliability and accuracy of this study’s results. However, in all samples, the immunolabeled CD31+ CNV lesions were larger than those measured by OCTA, and the size of the immunolabeled PDGFRβ+ pericyte-like scaffolds was larger than the CD31+ CNV lesions (S1 Table). Furthermore, we did not use electron microscopy to visualize the pericyte-like scaffold and without such ultrastructural assessment it is difficult to confirm the presence of myofibroblasts within the scaffold. While the sole use of immunohistochemistry may be insufficient to confirm the localization and identification of these cells, the result that the pericyte-like scaffold was stained by PDGFRβ, α-SMA and collagen type I would support our hypothesis that myofibroblasts in the pericyte-like scaffold play a role in the development of subretinal fibrosis. Furthermore, we obtained enface OCTA images between the deep retina and choroid using the semi-automatic mode, which assessed CNV without automatic removal of projection artifacts. Although we calculated the area of the CNV lesions by enface OCTA imaging confirming obvious projection artifacts, it is possible there is a tendency to overestimate the size of the CNV lesions by enface OCTA. Moreover, it is also possible there is a bias of field angle for OCTA images obtained by the only first author. This might affect area measurements of OCTA images.

## Conclusions

OCTA is a useful and noninvasive tool that allows for the visualization of CNV lesions without dye injection. However, OCTA images can have shadowing artifacts resulting from the presence of fibrosis and these artifacts may lead to an underestimation of CNV lesions in exudative AMD. When assessing CNV lesions by OCTA, it is necessary to confirm whether histological characteristics such as fibrosis result in imaging artifacts.

## Supporting information

S1 Fig OCT Image of wild-type C57BL/6J mouse. RNFL: retinal nerve fiber layer, GCL: ganglion cell layer, IPL: inner plexiform layer, INL: inner nuclear layer, OPL: outer plexiform layer, ONL: outer nuclear layer, RPE: retinal pigment epithelium.
(TIF)

S2 Fig Images of OCT, OCTA and immunolabeling experiments with frozen sections from laser-induced CNV at 5 days post-laser photocoagulation. (A) By enface OCTA, laser-induced CNV was observed as a hyperflow lesion surrounded by a dark halo (*; measured area: 900×900 μm). (B) By cross-sectional OCTA, corresponding to the horizontal blue line in S2 Fig A, blood flow was detected between the deep retina and the choroid (measured width: 900 μm). (C) By OCT, laser-induced CNV and the pericyte-like scaffold appeared as a subretinal hyper-reflective lesion (measured width: 900 μm). (D) Vessels were stained with CD31. The CNV lesion is inside a yellow circle. (E) The pericyte-like scaffold and pericytes were stained with PDGFRβ. The pericyte-like scaffold is inside a yellow circle. (F) Merged image of CD31 and PDGFRβ immunohistochemistry. The CNV lesion and the pericyte-like scaffold are inside a yellow circle. CNV was detected within the pericyte-like scaffold. Scale bar, 500 μm (D-F).
(TIF)

S1 Table Area measurements for all samples. In all samples, the areas measured by immunolabeling for the CD31+ CNV lesion were larger than those obtained by OCTA imaging. The size of the immunolabeled PDGFRβ+ pericyte-like scaffolds were larger than the CD31+ CNV lesions.
(TIF)
