# Supplementary figures and images for: Comparison between optical coherence tomography angiography and immunolabeling for evaluation of laser-induced choroidal neovascularization
Source: PLoS One. 2018 Aug 9;13(8):e0201958. doi: 10.1371/journal.pone.0201958 (PMC6084993; doi:10.1371/journal.pone.0201958)

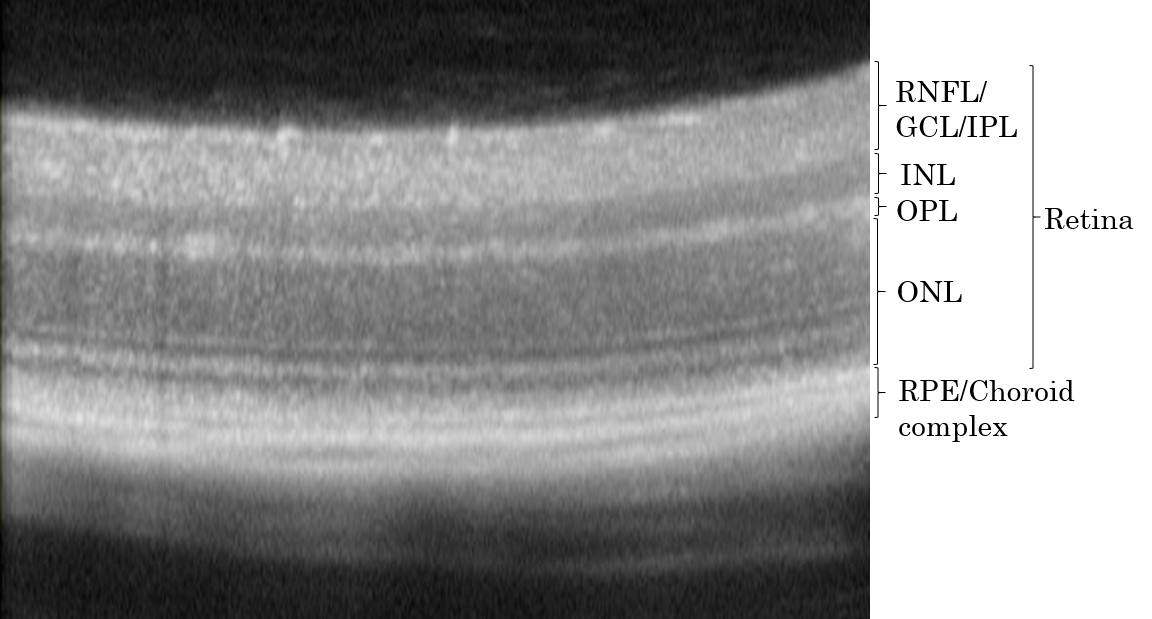

Supplement: S1 Fig — RNFL: retinal nerve fiber layer, GCL: ganglion cell layer, IPL: inner plexiform layer, INL: inner nuclear layer, OPL: outer plexiform layer, ONL: outer nuclear layer, RPE: retinal pigment epithelium. (TIF) [file pone.0201958.s001.tif]

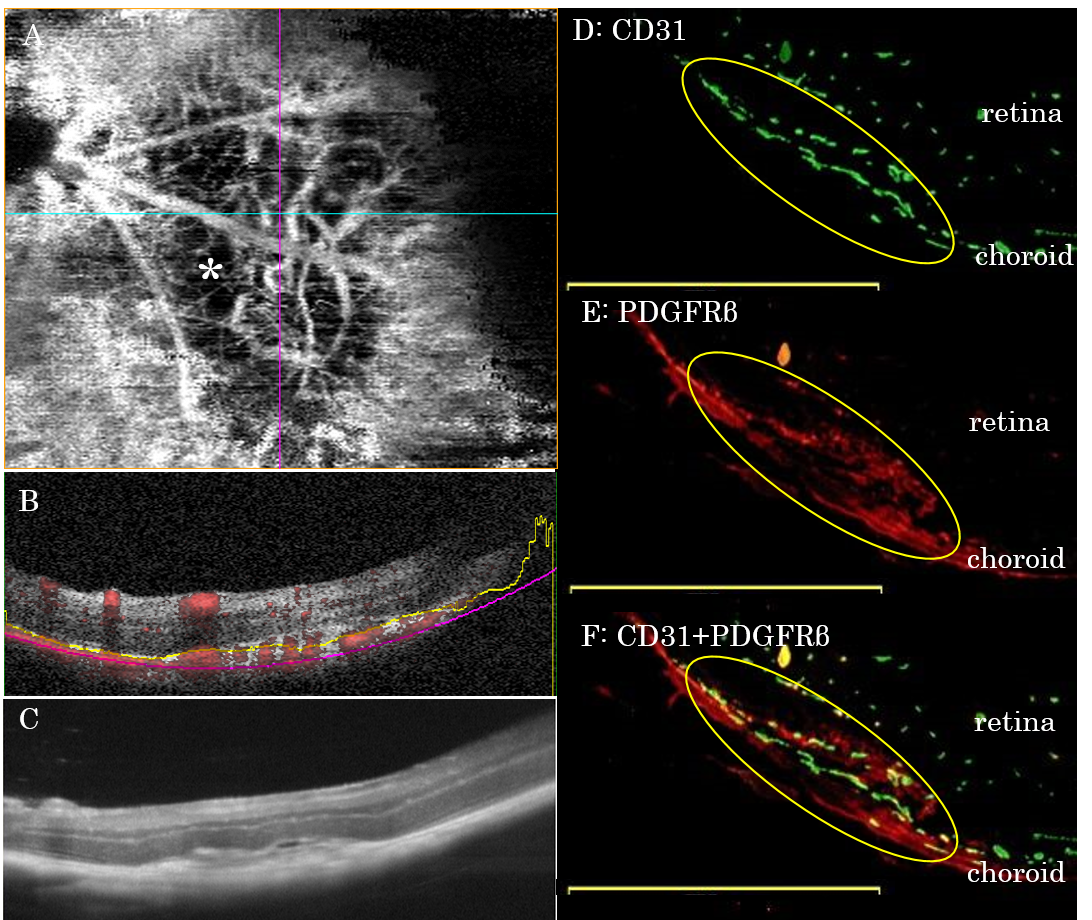

Supplement: S2 Fig — (A) By enface OCTA, laser-induced CNV was observed as a hyperflow lesion surrounded by a dark halo (*; measured area: 900×900 μm). (B) By cross-sectional OCTA, corresponding to the horizontal blue line in S2 Fig A, blood flow was detected between the deep retina and the choroid (measured width: 900 μm). (C) By OCT, laser-induced CNV and the pericyte-like scaffold appeared as a subretinal hyper-reflective lesion (measured width: 900 μm). (D) Vessels were stained with CD31. The CNV lesion is inside a yellow circle. (E) The pericyte-like scaffold and pericytes were stained with PDGFRβ. The pericyte-like scaffold is inside a yellow circle. (F) Merged image of CD31 and PDGFRβ immunohistochemistry. The CNV lesion and the pericyte-like scaffold are inside a yellow circle. CNV was detected within the pericyte-like scaffold. Scale bar, 500 μm (D-F). (TIF) [file pone.0201958.s002.tif]

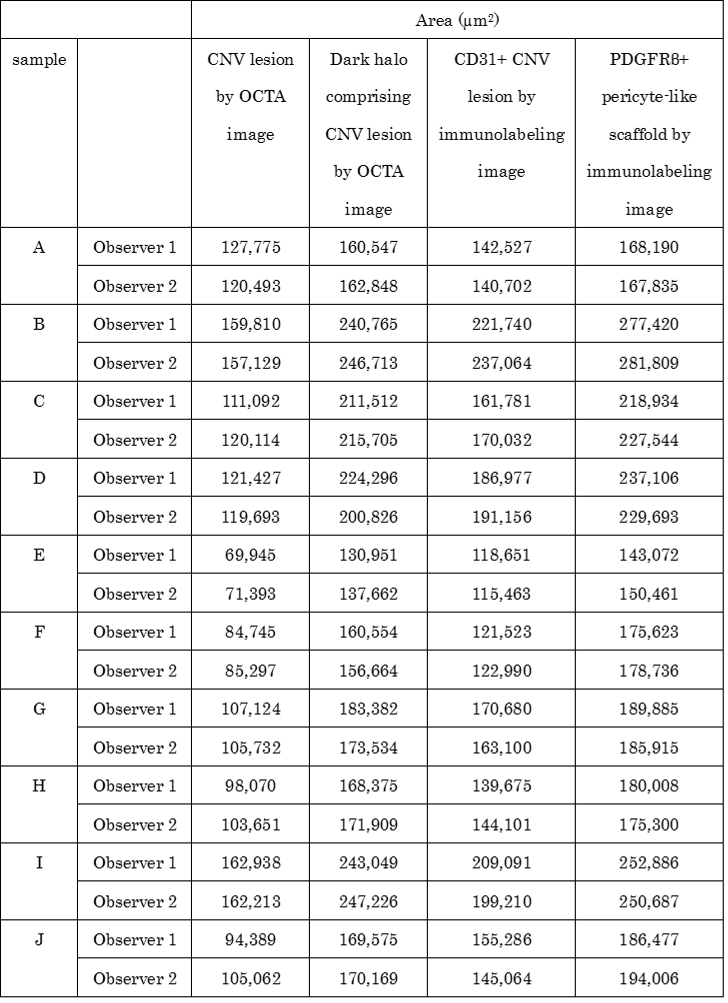

Supplement: S1 Table — In all samples, the areas measured by immunolabeling for the CD31+ CNV lesion were larger than those obtained by OCTA imaging. The size of the immunolabeled PDGFRβ+ pericyte-like scaffolds were larger than the CD31+ CNV lesions. (TIF) [file pone.0201958.s003.tif]
